# How accurately can surface markers be placed on bony landmarks of the foot?

**DOI:** 10.1186/1757-1146-5-S1-O40

**Published:** 2012-04-10

**Authors:** Alpesh Kothari, Julie Stebbins, Jessica Leitch, Amy Zavatsky

**Affiliations:** 1 Nuffield Orthopaedic Centre, Oxford, OX3 7LD, UK; 2 Department of Engineering Science, University of Oxford, Oxford OX1 3PJ, UK

## Background

The use of multi-segment foot models is becoming increasingly popular during clinical gait analysis. While numerous studies have established the repeatability of these models, the accuracy is more difficult to determine since measuring motion of the bones is a challenging task. One assumption influencing model accuracy is that surface markers can be placed precisely over palpated, bony landmarks. The aim of this study is to test this assumption by assessing marker placement using CT scans.

## Materials and methods

Twenty female subjects (forty feet) participated in this study. All subjects had ECG electrodes attached to their lower limbs according to the positions required by the Oxford Foot Model [1]. Positioning was performed by a single tester on all subjects. Subjects lay supine in the CT scanner, in a semi-weight-bearing position using a custom-built rig. The anatomical landmarks and the positions of the markers were identified on the scans using a pre-defined protocol. Intra- and inter-rater reliability were assessed. Marker placement accuracy was determined by assessing relevant components of the distance between markers and bony landmarks.

## Results

**Figure 1:**
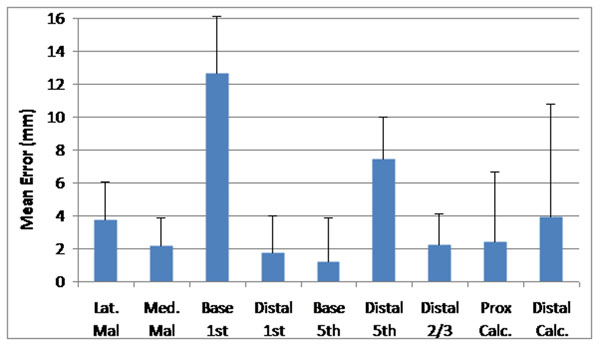
Mean error (mm) across all feet for all markers

Good intra- and inter-rater reliability was demonstrated for identifying markers on the CT images (Table 1). The average distance between bony landmarks and marker positions differed according to position on the foot (Figure 1). The mean error was lowest for the base of 5^th^ metatarsal marker (1.2 mm) and highest for the base of 1^st^ metatarsal (12.7 mm). There was a systematic offset for this marker, due to slight differences in definition for placing the marker on the skin, and identifying the bony landmark on CT images. Of the nine marker positions analysed, seven markers had a mean error of less than 5 mm.

**Table 1:** Reliability for identifying landmarks

	95% confidence interval
Intra-observer (n=3)	0.19 mm - 0.37 mm
Inter-observer (n=3)	0.21 mm - 0.57 mm

## Conclusions

Surface markers can be placed accurately over bony landmarks on the foot; however, some positions can be more precisely palpated than others. This should be taken into account when interpreting results from multi-segment foot models.
